# Retreatment of a Maxillary Lateral Incisor With Two Separate Root Canals Confirmed With Cone Beam Computed Tomography

**DOI:** 10.14740/jocmr2154w

**Published:** 2015-05-08

**Authors:** Seda Aydemir, Dilek Helvacioglu-Yigit, Alper Sinanoglu, Emre Ozel

**Affiliations:** a Department of Endodontics, Faculty of Dentistry, University of Kocaeli, Kocaeli, Turkey; b Department of Oral and Maxillofacial Radiology, Faculty of Dentistry, University of Kocaeli, Kocaeli, Turkey; c Department of Restorative Dentistry, Faculty of Dentistry, University of Kocaeli, Kocaeli, Turkey

**Keywords:** Cone-beam computed tomography, Endodontic retreatment, Extra root canal, Maxillary lateral incisor

## Abstract

The purpose of this report is to present a rare case of a maxillary lateral incisor exhibiting two separate root canals confirmed by cone-beam computed tomography (CBCT). A 65-year-old female patient with an esthetic complaint regarding her maxillary left lateral incisor was referred to our clinic. During a radiographical examination, an endodontically treated root canal and an extra root canal with an apical lesion were observed. The retreatment was performed. CBCT findings confirmed the root canal mophology of the maxillary left lateral with two distinct canals. We conclude that the CBCT imaging is an adjunctive tool for better assessment of complex root canal systems.

## Introduction

It is well known that the success of endodontic therapy depends on the complete cleaning and shaping of the root canal systems, three-dimensional obturation, and proper coronary sealing. To obtain successful root canal treatment, knowledge of the morphology of the root canal system as well as its variations is essential. The maxillary left lateral incisor is known to display anatomical variations.

The two-canal morphology of the mandibular premolars is rarely considered in diagnostic radiography. The separation of the secondary canal with an acute angle means the second canal remains undiagnosed both by radiography and tactile examination [1]. Undetected canals are the cause of 42% of root canal retreatments [2]. Cone-beam computed tomography (CBCT) is a non-invasive technique to determine the existence of an extra root canal. It reveals the true nature of macrostructure three-dimensionally and its curvature and angulation [3]. CBCT presents the precise position of the extra root canal; hence, it helps to track the curvature and prevents iatrogenic errors that might occur in relation to canal curvature such as instrument separation, perforation, and ledge formation.

The purpose of this report is to present a rare case of maxillary lateral incisor exhibiting two separate root canals that was confirmed by CBCT findings.

## Case Report

**Figure 1:**
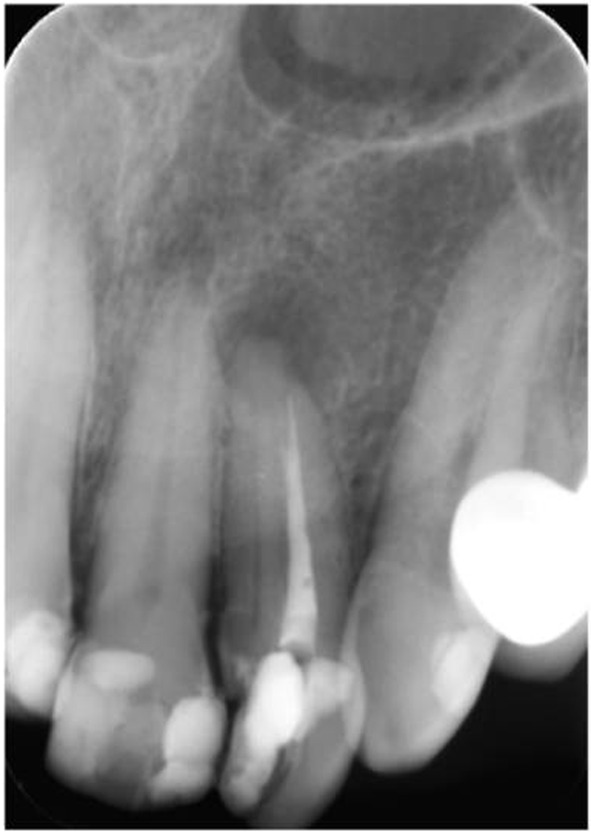
Preoperative periapical radiography.

**Figure 2:**
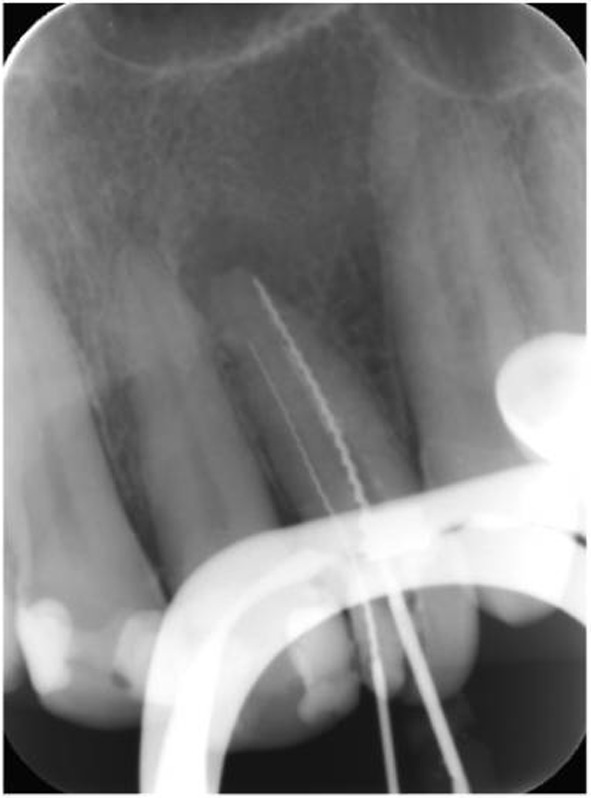
Working length determination.

A 65-year-old female patient with an esthetic complaint regarding her maxillary left lateral incisor was referred to our clinic. During a radiographic examination, an endodontically treated root canal and an extra root canal with an apical lesion were observed (Fig. 1). The existing root filling was removed using ProTaper Universal retreatment files (Dentsply Maillefer, Ballaigues, Switzerland). D1, D2 and D3 retreatment files were used in the crown-down technique with a low torque motor (VDW Silver; VDW). The missing root canal was located and the working length of both root canals was measured with an apex locator Raypex 6 (VDW, Munich, Germany) and confirmed with a periapical radiograph (Fig. 2).

**Figure 3:**
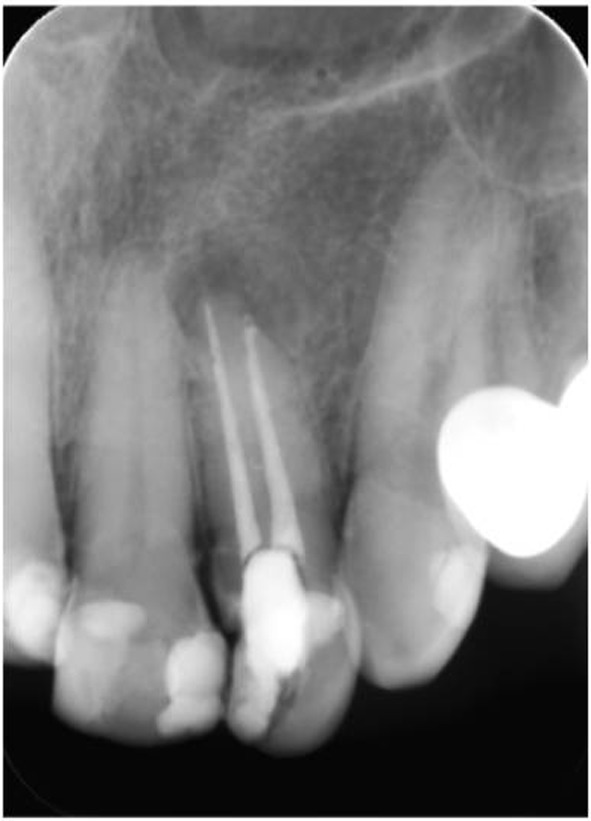
Postoperative periapical radiography.

The root canals were then shaped with ProTaper Universal instruments (Dentsply Maillefer, Ballaigues, Switzerland) with a sequence of SX, S1, S2, F1 and F2 according to the manufacturer’s instructions. The root canals were lubricated with MM-EDTA cream (Micro-Mega, Besancon, France), and irrigated with 2 mL of 2.5% NaOCl between instruments and then obturated using cold lateral compaction of gutta-percha (Diadent, Seoul, South Korea) with AH Plus sealer (Dentsply De Trey GmbH, Konstanz, Germany) (Fig. 3).

The patient was also referred to the Department of Oral and Maxillofacial Radiology for CBCT evaluation of her maxilla for implant site assessment (Planmeca ProMax 3D Max, Planmeca Oy, Helsinki, Finland). Axial, panoramic and cross-sectional images were reconstructed with a slice thickness of 1 mm. CBCT findings confirmed the root canal morphology of the maxillary left lateral with two separate canals.

**Figure 4:**
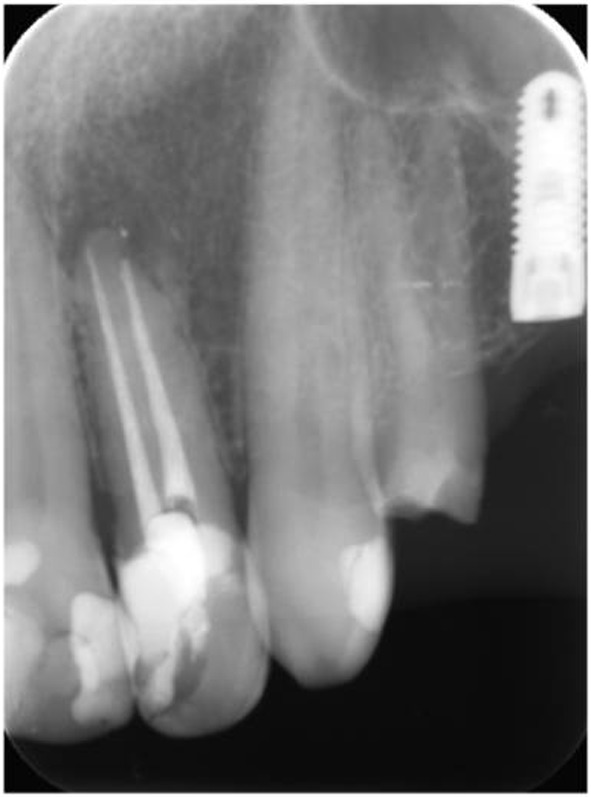
Periapical radiography after 18 months follow-up.

Following the root canal treatment, the tooth was etched with 37% phosphoric acid, and restored with an adhesive system (Single Bond Universal Adhesive, 3M ESPE, USA) and nanocomposite (Filtek Ultimate Universal Restorative, 3M ESPE, USA). Finishing and polishing procedures were performed by discs and burs. A recall was carried out at the end of 18 months (Fig. 4).

**Figure 5:**
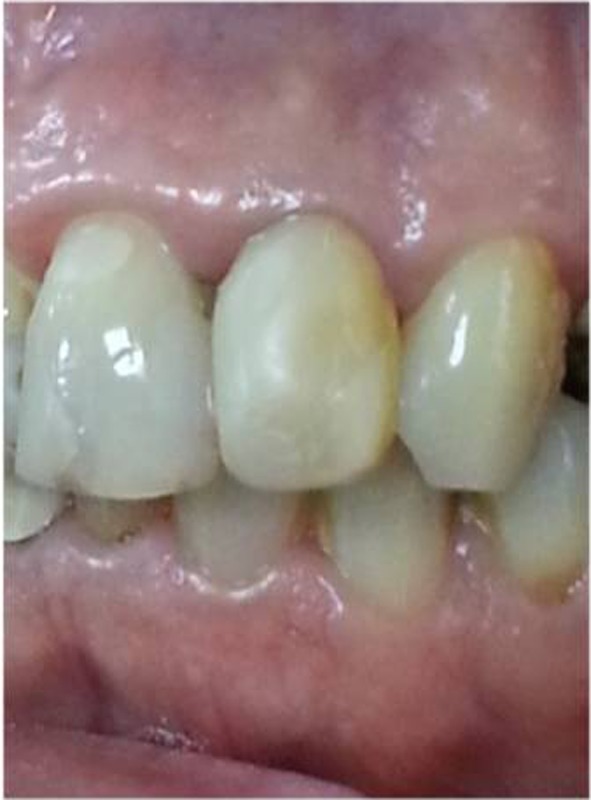
Clinical view after 18 months follow-up.

During the recall, composite restoration was found successful according to the criteria of the United States Public Health Service (USPHS) (Table 1) in terms of retention, color match, secondary caries, anatomic form, marginal adaptation and surface texture (Fig. 5).

**Table 1:** United States Public Health Service (USPHS) Criteria

Category	Scores	Criteria
Retention	Alfa	No loss of restorative material
	Charlie	Any loss of restorative material
Color match	Alfa	Matches tooth
	Bravo	Acceptable mismatch
	Charlie	Unacceptable mismatch
Secondary caries	Alfa	No caries present
	Charlie	Caries present
Anatomic form	Alfa	Continuous
	Bravo	Slight discontinuity, clinically acceptable
	Charlie	Discontinuous, failure
Marginal adaptation	Alfa	Closely adapted, no detectable margin
	Bravo	Detectable margin, clinically acceptable
	Charlie	Marginal crevice, clinical failure
Surface texture	Alfa	Enamel-like surface
	Bravo	Surface rougher than enamel, clinically acceptable
	Charlie	Surface unacceptable rough

## Discussion

This report describes a rare case of a maxillary lateral incisor tooth that had two separate root canals in one root yet did not exhibit any morphological anomaly of the crown. A maxillary lateral incisor tooth most frequently exhibits a single root with a single canal [4]. However, there are a few case reports describing maxillary lateral incisor teeth with two canals [5-7], three canals [8, 9], and even four canals [10]. Many of these cases omanifest clinically as gemination, fusion, concrescence, or dens invaginatus since maxillary lateral incisors are often located at the site of high embryological risk [11-13]. In the present case, none of these abnormalities (i.e., gemination, fusion, concrescence, or dens invaginatus) was observed.

The probable etiology for unilateral accessory roots is traumatic injury of primary teeth. Root duplication may occur following intrusive luxation of primary teeth, resulting in a traumatic division of the cervical loop and thus formation of two separate roots. In these cases, a mesial and a distal root can be detected on the radiograph [14].

An emerging technology, CBCT has the ability to assess an area of interest in three dimensions, thereby eliminating the superimposition problem of conventional radiographic imaging. CBCT provides detailed and three-dimensional information concerning extra canals, apical deltas, and canal type. It provides accurate measurement potential in all aspects of root canal system, and in brief, a three-dimensional perspective of root canal anatomy [15, 16]. But use of CBCT must be limited. It may be justified for selected cases, such as when intraoral radiographs provide equivocal information on root canal anatomy or the information is inadequate for planning treatment, as is the case with multi-rooted teeth.

**Figure 6:**
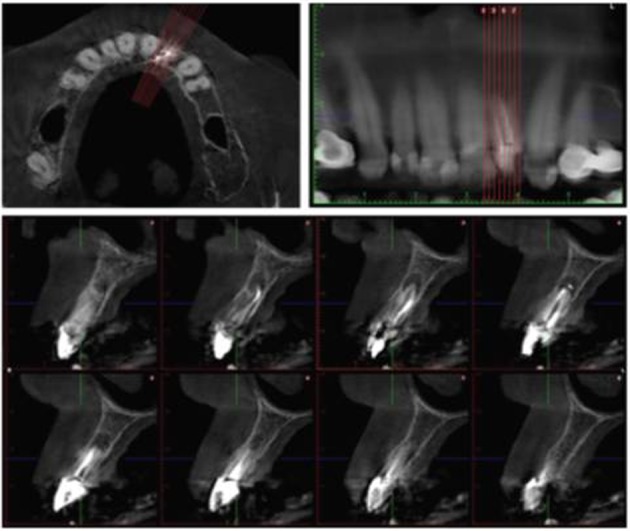
Axial, panoramic and cross-sectional reconstruction with a slice thickness at 1 mm.

In the present case, the CBCT scan was not performed for endodontic purposes. The CBCT findings confirmed two separate canals in the maxillary left lateral (Fig. 6). Eighteen months after the procedure, the radiographical examination showed a decrease in the size of the periapical lesion. In addition, the composite restoration scored alpha for all USPHS criteria during the 18-month recall. The follow-up demonstrated a clinically asymptomatic and adequately functional tooth.

The clinician should be aware of such abnormalities in root morphology. CBCT imaging is an adjunctive tool for better assessment of complex root canal systems.
